# Analytical and Clinical Evaluation of “AccuPower SARS-CoV-2 Multiplex RT-PCR kit (Bioneer, South Korea)” and “Allplex 2019-nCoV Assay (Seegene, South Korea)” for SARS-CoV-2 RT-PCR Diagnosis: Korean CDC EUA as a Quality Control Proxy for Developing Countries

**DOI:** 10.3389/fcimb.2021.630552

**Published:** 2021-06-10

**Authors:** Byron Freire-Paspuel, Miguel Angel Garcia-Bereguiain

**Affiliations:** One Health Research Group, Universidad de Las Americas, Quito, Ecuador

**Keywords:** SARS-CoV-2, RT-PCR, Bioneer, Seegene, developing countries

## Abstract

**Background:**

Multiple RT-qPCR kits are available in the market for SARS-CoV-2 diagnosis, some of them with Emergency Use Authorization (EUA) by FDA or their country of origin agency, but many of them lack of proper clinical evaluation.

**Objective:**

We evaluated the clinical performance of two Korean SARS-CoV-2 RT-PCR kits available in South America, AccuPower SARS-CoV-2 Multiplex RT-PCR kit (Bioneer, South Korea) and Allplex 2019-nCoV Assay (Seegene, South Korea), for RT-qPCR SARS-CoV-2 diagnosis using the CDC protocol as a gold standard.

**Results:**

We found strong differences among both kits clinical performance and analytical sensitivity; while the Allplex 2019-nCoV Assay has sensitivity of 96.5% and an estimated limit of detection of 4,000 copies/ml, the AccuPower SARS-CoV-2 Multiplex RT-PCR kit has a sensitivity of 75.5% and limit of detection estimated to be bigger than 20,000 copies/ml.

**Conclusions:**

AccuPower SARS-CoV-2 Multiplex RT-PCR kit and Allplex 2019-nCoV Assay are both made in South Korea but EUA by Korean CDC was only granted to the later. Our results support that Korean CDC EUA should be considered as a quality control proxy for Korean SARS-CoV-2 RT-PCR kits prior to importation by developing countries to guarantee high sensitivity diagnosis.

## Introduction

The COVID-19 pandemic has affected public health systems worldwide, challenging patient care quality and surveillance and control success. Moreover, to guarantee the quality of SARS-CoV-2–related diagnosis tools was compromised under an emergency use authorization (EUA) scenario. Multiple *in vitro* RT-qPCR diagnosis kits are available on the market for the detection of SARS-CoV-2, with EUA from the U.S. Food & Drug Administration (FDA) or other international agencies like Chinese CDC or Korean CDC, or without EUA and limited validations studies made by manufacturers. The CDC-designed FDA EUA 2019-nCoV CDC kit (IDT, USA) is based on N1 and N2 gene targets to detect SARS-CoV-2 and RNase P as an RNA extraction quality control, it is considered a gold standard worldwide for SARS-CoV-2 RT-PCR diagnosis (CDC, 2020; Nalla et al., 2020; Rhoads et al., 2020; Xiaoyan et al., 2020). Our lab has recently published a highly sensitive adaptation of the CDC protocol on a triplex assay format that is time and cost effective (Freire-Paspuel and Garcia-Bereguiain, 2021a).

Among the commercial kits available in South America for SARS-CoV-2 diagnosis, several are made in South Korea. For instance, Allplex 2019-nCoV Assay (Seegene, South Korea) is a multiplex SARS-CoV-2 RT-qPCR kit for E, N and RdRp genes that holds both FDA EUA and Korean CDC EUA. On the other hand, AccuPower SARS-CoV-2 Multiplex RT-PCR kit (Bioneer, South Korea) is also a multiplex SARS-CoV-2 RT-qPCR assay for E, N, and RdRp genes, which are not included neither in the FDA EUA list nor in the Korean CDC EUA list (FDA, 2020; Hong et al., 2020). Moreover, we have previously reported that other South Korean SARS-CoV-2 RT-PCR and RT-LAMP kits lacking Korean CDC EUA did not accomplish the clinical performance and analytical sensitivity described at the manufacturer’s manual (Freire-Paspuel et al., 2020a; Freire-Paspuel and Garcia-Bereguiain, 2020; Freire-Paspuel and Garcia-Bereguiain, 2021b).

We herein present a comparison of the analytical and clinical performance of AccuPower SARS-CoV-2 Multiplex RT-PCR kit (Bioneer, South Korea) and Allplex 2019-nCoV Assay (Seegene, South Korea) for SARS-CoV-2 RT-qPCR diagnosis from nasopharyngeal samples.

## Materials and Methods

### Study Design

89 clinical specimens (nasopharyngeal swabs collected on 0.5 ml TE pH 8 buffer) were included in this study, coming from individuals selected for SARS-CoV-2 surveillance at the laboratory of “Universidad de Las Américas” in Quito (Ecuador). Also, negative controls (TE pH 8 buffer) were included as control for carryover contamination, one for each set of RNA extractions.

### RNA Extraction

All the samples were processed with the same RNA extraction kit “AccuPrep Viral RNA extraction kit” (Bioneer, South Korea), and the same RNA extraction was used for the three RT-PCR protocols. A maximum of a cycle of −80°C frozen/thawed was applied for some samples that could not be processed within the same day that the CDC protocol with the other commercial kits. We have also tested in our lab that RNase P values do not vary after cycles of frozen/thawed as no statistically significant differences were found for RNase P Ct values when an “inconclusive” (amplification of only N1 or N2 gene targets) sample has to be repeated. So, we can certainly assure that no RNA degradation happens for the experimental conditions described in our study.

### RT-qPCR for SARS-CoV-2 Diagnosis Using the CDC RT-PCR Protocol

All the samples included in the study were tested following an adapted version of the CDC protocol: (a) using Accupower Viral RNA extraction kit (Bioneer, South Korea) as an alternate RNA extraction method; (b) using CFX96 BioRad instrument (Freire-Paspuel et al., 2020a; Freire-Paspuel and Garcia-Bereguiain, 2020; Freire-Paspuel et al., 2020; Freire-Paspuel et al., 2020b; Freire-Paspuel et al., 2020; Freire-Paspuel and Garcia-Bereguiain, 2021b; Freire-Paspuel et al., 2021; Ortiz-Prado et al., 2021); (c) using a N1/N2/RNaseP multiplex assay (Freire-Paspuel and Garcia-Bereguiain, 2021a). The criteria for positivity was a Ct ≤ 40 for N1 and N2 targets simultaneously (CDC, 2020; Xiaoyan et al., 2020).

### RT-qPCR for SARS-CoV-2 Diagnosis Using AccuPower SARS-CoV-2 Multiplex RT-PCR Kit

Same RNA extractions from all the samples included on the study were tested using AccuPower SARS-CoV-2 Multiplex RT-PCR kit, following the manufacturer’s manual (Ref: SCVM-2112 Lot: 200231G). Accordingly, a sample is considered positive if both E and RrRp-N targets amplify, or just only RdRp-N, with Ct ≤ 35 and Ct ≤ 34, respectively. If only E gene target amplify, the sample is considered “presumptive positive”. Although a master mix including internal control was prepared for each batch of tested samples and the internal control amplified in most of the samples, we only considered a CDC protocol SARS-CoV-2 positive sample as negative sample for AccuPower kit if the internal control amplified. So, for sensitivity calculation we excluded “invalid samples” (See **Supplementary Table 1**): SARS-CoV-2 positive samples according to the CDC protocol where neither E/RdRp-N targets viral targets nor the internal control amplified. So far, internal control amplification is not related to the quality of the sample as the RNaseP target on the CDC protocol, but to make our evaluation as fair as possible we excluded those samples defined as invalid.

### RT-qPCR for SARS-CoV-2 Diagnosis Using Allplex 2019-nCoV Assay

Same RNA extractions from all the samples included on the study were tested using Allplex 2019-nCoV Assay, following the manufacturer’s manual (Ref: RP10243X Lot: RP4520GB6). Accordingly, a sample is considered positive if at least N or RdRp targets amplify with Ct ≤ 40. If only E gene target amplify with Ct ≤ 40, the sample is considered “presumptive positive”. Also, if the internal positive control has a Ct > 40 in the absence of E, RdRp or N amplification in a SARS-CoV-2 positive sample for to the CDC protocol, the sample is considered invalid.

### Analytical Sensitivity

Limit of detection (LoD) was performed using the commercially available 2019-nCoV N-positive control (IDT, USA); provided at 200,000 genome equivalents/ml, it was used for calibration curves to obtain the viral loads of the samples. Viral loads can be expressed as copies/µl of RNA extraction or copies/ml of sample; the conversion factor is 200, as 0.2 ml of sample is used for RNA extraction and 40 µl is used as final elution volume of RNA extraction.

## Results

### Clinical Performance and Estimation of Limit of Detection of Allplex 2019-nCoV Assay Using the CDC RT-PCR Protocol as a Gold Standard

89 samples were tested for SARS-CoV-2 following both Allplex 2019-nCoV Assay and CDC RT-PCR protocol, as described in *Materials and Methods*. For the CDC protocol, 57 samples tested positive and 32 samples tested negative (**Table 1** and **Supplementary Table 1**). 30 out of 32 samples tested negative for the CDC protocol were also SARS-CoV-2 negative for Allplex 2019-nCoV Assay, so the specificity obtained in our study was 93.75%. The two “false positive” samples had Ct values > 36 (Samples 58 and 59 at **Supplementary Table 1**).

**Table 1:** Clinical performance of AccuPower SARS-CoV-2 Multiplex RT-PCR kit and Allplex 2019-nCoV Assay using the CDC protocol as a gold standard (% values: sensitivity).

RT-PCR kit	Positive samples (including “presumptive positive: samples)	False-negative samples	Total SARS-CoV-2 positive samples
**Allplex 2019-nCoV Assay**	**55 (96.5%)**	**2**	**57**
**AccuPower SARS-CoV-2 Multiplex RT-PCR kit**	**37 (75.5%)**	**12**	**49**

Only SARS-CoV-2 positive samples included on the study are detailed.

For the 57 SARS-CoV-2–positive samples for the CDC RT-PCR protocol, 55 samples tested also positive for Allplex 2019-nCoV Assay, resulting a sensitivity of 96.5% (**Table 1** and **Supplementary Table 1**). The viral loads for the two SARS-CoV-2 positive samples that tested negative for Allplex 2019-nCoV Assay were as low as 12.8 and 5.15 copies/µl (< 3000 copies/ml).

As the limit of detection (LoD) is defined as the lowest viral load in which all replicates are detected (100% sensitivity), our data indicate that the LoD for Allplex 2019-nCoV assay should be around 20 copies/µl of RNA extraction (4,000 viral RNA copies/ml of sample), as no failure to detect SARS-CoV-2–positive samples above this threshold was found (**Supplementary Table 1**).

### Clinical Performance and Estimation of Limit of Detection of Using AccuPower SARS-CoV-2 Multiplex RT-PCR Kit Using the CDC RT-PCR Protocol as a Gold Standard

89 samples were tested for SARS-CoV-2 following both using AccuPower SARS-CoV-2 Multiplex RT-PCR kit and the CDC RT-PCR protocol, as described on the methods. For the CDC protocol, 57 samples tested positive and 32 samples tested negative (**Table 1** and **Supplementary Table 1**). None of the negative samples for the CDC RT-PCR protocol were positive for AccuPower SARS-CoV-2 Multiplex RT-PCR kit, so the specificity obtained in our study was 100%.

From the 57 samples that tested positive for the CDC RT-PCR protocol, eight of them were invalid for the AccuPower SARS-CoV-2 Multiplex RT-PCR kit, as neither the viral gene targets nor the internal positive control amplified (see **Supplementary Table 1**). For the remaining 49 SARS-CoV-2 positive samples, 35 samples were true positive and two were “presumptive positive” (only amplification of E gene target) for AccuPower SARS-CoV-2 Multiplex RT-PCR kit. Considering only true-positive samples, the sensitivity was 71.4% (35/49), but up to 75.5% (37/49) if we also included “presumptive positive” samples for AccuPower SARS-CoV-2 Multiplex RT-PCR kit (**Table 1** and **Supplementary Table 1**).

As the limit of detection (LoD) is defined as the lowest viral load in which all replicates are detected (100% sensitivity), our data indicate that the LoD for AccuPower SARS-CoV-2 Multiplex RT-PCR kit should be bigger than 90 copies/µl of RNA extraction (18,000 viral RNA copies/ml of sample), as even two SARS-CoV-2 positives samples with viral loads of 203 and 90.4 copies/µl failed to be positive for AccuPower SARS-CoV-2 Multiplex RT-PCR kit. The sensitivity obtained for that viral load threshold was 94.3% (33/35) (see **Supplementary Table 1**).

## Discussion

Although the main limitations of our study is the sample size (89 specimens) and that a single lot of each of the kits was used for the evaluation, our results support that Allplex 2019-nCoV Assay had a great performance in terms on sensitivity compared to the CDC RT-PCR protocol, with values up to 96.5%. However, we found a relatively low specificity of 93.75%; as it has been previously reported that no cross reactivity with other respiratory viruses happened for Allplex 2019-nCoV Assay (Farfour et al., 2020), further studies are needed to set a most accurate specificity value as we only included 32 negative samples in ours. As we have described on the results, we could estimate de LoD of Allplex 2019-nCoV Assay on approximately 4,000 viral RNA copies/ml of sample which is similar to the LoD of 4,167 copies/ml detailed at manufacturer’s manual. This LoD is acceptable for a reliable SARS-CoV-2 diagnosis considering the viral load frequency population distributions (Kleiboeker et al., 2020; Lavezzo et al., 2020).

Although AccuPower SARS-CoV-2 Multiplex RT-PCR kit has a great specificity (100%), our data showed that this kit had a low clinical performance in terms on sensitivity compared to the CDC RT-PCR protocol, with values of 75.5% and 71.4% depending if we consider “presumptive positive” (E gene amplification only) samples as true SARS-CoV-2 positive or not. Moreover, as we have described on the results, we could estimate de LoD of AccuPower SARS-CoV-2 Multiplex RT-PCR kit to be above on 18,000 viral RNA copies/ml of sample, as two SARS-CoV-2 positive samples above this threshold failed to test positive, resulting a sensitivity of 94.3% for that viral load threshold, below the 100% expected for the LoD. AccuPower SARS-CoV-2 Multiplex RT-PCR kit manufacturer’s manual reported a LoD of six copies/µl, equivalent to 1,200 copies/ml on our experimental conditions, but according to our data the LoD would be higher.

In **Table 2**, analytical parameters and other characteristics for Allplex 2019-nCoV Assay and AccuPower SARS-CoV-2 Multiplex RT-PCR kits are summarized. As we have detailed on the introduction, only Allplex 2019-nCoV Assay has both FDA EUA and Korean CDC EUA. Considering the better analytical sensitivity for Allplex 2019-nCoV Assay compared to AccuPower SARS-CoV-2 Multiplex RT-PCR kit, our study suggests that a good public policy for developing countries like Ecuador would be to use Korean CDC EUA as a quality control proxy to allow the importation of South Korean SARS-CoV-2 RT-PCR kits. Actually, we have already published other clinical evaluations for South Korean RT-PCR and RT-LAMP kits that endorse this same idea, as neither nCoV-QS (MiCo BioMed, South Korea) and AccuPower SARS-CoV-2 Real Time RT-PCR kit (Bioneer, South Korea) RT-PCR kits nor Isopollo COVID-19 detection kit (M Monitor, South Korea) RT-LAMP kit have Korean CDC EUA; and the three of them had a low clinical performance and a LoD much higher of what is indicated by the manufacturers. So far, we expressed our concern toward local regulatory agencies that allow these companies to export SARS-CoV-2 diagnosis kits to Ecuador and other South American countries despite their products lack of clinical use authorization in South Korea

**Table 2:** Comparison of CDC-RT-PCR protocol, AccuPower SARS-CoV-2 Multiplex RT-PCR and Allplex 2019-nCoV Assay kits.

SARS-CoV-2 RT-PCR kit (company/country)	Viral Gene Targets	Limit of detection observed (promised by manufacturer)	EUA
**2019-nCoV CDC EUA (IDT, USA)**	N1, N2	1,000 viral copies/ml	FDA
**Allplex 2019-nCoV Assay (Seegene, South Korea)**	E, N, RdRp	4,000 viral copies/ml (4,167 viral copies/ml)	FDA/K-CDC
**AccuPower SARS-CoV-2 Multiplex RT-PCR kit (Bioneer, South Korea)**	E, N/RdRp*	>20,000 viral copies/ml (1,200 viral copies/ml)	NONE

The limit of detection of AccuPower SARS-CoV-2 Multiplex RT-PCR detailed on manufacturer’s manual is six copies/µl, equivalent to 1200 copies/mL, on our experimental conditions.

*AccuPower SARS-CoV-2 Multiplex RT-PCR included a target called “SARS-CoV-2 gene” that includes RdRp and N gene; EUA, emergency use authorization; FDA, Federal Drug Administration; K-CDC, Korean CDC.

Considering the worldwide high demand of reagents for SARS-CoV RT-qPCR diagnosis, supplies shortage is a fact, EUA are necessary and regulations need to be more flexible. However, under this scenario, the role of Academia to carry out independent clinical performance and analytical sensitivity evaluations is crucial to guarantee the quality of the supplies in the market for every country in the world, particularly for developing countries usually lacking of reliable regulatory agencies. It is a matter of global justice and human rights.

## Data Availability Statement

The original contributions presented in the study are included in the article/**Supplementary Material**. Further inquiries can be directed to the corresponding author.

## Ethics Statement

All samples have been submitted for routine patient care and diagnostics. Ethics approval was not sought because the study involves laboratory validation of test methods and the secondary use of anonymous pathological specimens that falls under the category ‘exempted’ by Comité de Etica para Investigación en Seres Humanos from “Universidad de Las Américas”.

## Author Contributions

BF-P and MG-B performed the experiments, analyzed the data, and wrote the manuscript. All authors contributed to the article and approved the submitted version.

## Funding

This study was funded by Universidad de Las Americas (Quito, Ecuador).

## Conflict of Interest

The authors declare that their affiliation institution “Universidad de Las Américas” is involved in the commercialization of “ECUGEN SARS-CoV-2 RT-qPCR kit”. However, that kit was not used in the present study.
