# Supplementary material for: Analytical and Clinical Evaluation of “AccuPower SARS-CoV-2 Multiplex RT-PCR kit (Bioneer, South Korea)” and “Allplex 2019-nCoV Assay (Seegene, South Korea)” for SARS-CoV-2 RT-PCR Diagnosis: Korean CDC EUA as a Quality Control Proxy for Developing Countries
Source: Front Cell Infect Microbiol. 2021 Jun 10;11:630552. doi: 10.3389/fcimb.2021.630552 (PMC8223252; doi:10.3389/fcimb.2021.630552)
Supplement: Supplementary file 1 [file DataSheet_1.pdf]

Supplementary Table 1. Ct values and viral loads (viral RNA copies/uL of RNA extraction solution) for samples processed with the CDC RT-PCR protocol, AccuPower SARS-CoV-2 Multiplex RT-PCR and Allplex 2019-nCoV Assay kits (P.Positive: presumptive positive sample, only E gene amplified; Invalid samples: SARS-CoV-2 positive samples according to the CDC protocol where neither the viral targets nor the internal control amplified for Seegene and Bioneer protocols. Although a master mix including internal control was prepared for a batch of samples and most of the samples were positive for the internal control, we only considered a CDC protocol positive sample as negative sample for Bioneer if the internal control amplified).

		CDC Protocol					Allplex (SeeGene) Protocol					Accupower Multiplex (Bioneer) Protocol			
N	**ID**	Viral Load (c/µL)	N1 Ct	N2 Ct	RP Ct	Result	E Ct	N Ct	RdRP Ct	IC Ct	Result	E Ct	N/RdRP Ct	IPC Ct	Result
1	**10647**	3,77E+06	15,1	16,8	20,8	Positive	17,2	16,3	19,1	NA	Positive	18,2	15,6	NA	Positive
2	**15988**	1,97E+06	16,1	16,6	22,9	Positive	17,4	19,0	21,2	NA	Positive	18,0	17,5	38,7	Positive
3	**16139**	1,83E+06	16,2	17,1	22,1	Positive	17,2	19,1	20,7	NA	Positive	18,3	18,0	36,5	Positive
4	**16096**	1,05E+06	17,0	18,0	26,2	Positive	17,6	19,5	21,0	NA	Positive	18,9	18,6	34,3	Positive
5	**16156**	8,62E+05	17,3	18,4	23,9	Positive	18,8	20,3	21,4	NA	Positive	19,2	19,1	NA	Positive
6	**16058**	4,35E+05	18,4	19,3	24,3	Positive	19,4	20,8	22,8	NA	Positive	19,5	19,4	33,1	Positive
7	**15959**	3,25E+05	18,8	20,2	24,8	Positive	19,3	21,5	22,9	NA	Positive	20,5	20,8	37,5	Positive
8	**16203**	1,22E+05	20,4	21,5	21,4	Positive	21,9	23,4	24,5	NA	Positive	20,8	20,6	34,8	Positive
9	**10646**	8,83E+04	20,9	22,2	24,2	Positive	22,0	22,9	24,2	41,0	Positive	9,4	9,4	9,6	Positive
10	**16933**	8,41E+04	20,9	22,1	26,7	Positive	21,3	22,9	23,7	40,1	Positive	22,2	22,3	33,6	Positive
11	**15964**	4,00E+04	22,1	23,0	23,2	Positive	23,5	24,6	26,1	NA	Positive	24,6	24,1	14,9	Positive
12	**17894**	2,43E+04	22,8	23,7	26,4	Positive	24,4	25,8	28,0	31,3	Positive	24,2	23,6	29,4	Positive
13	**15965**	1,46E+04	23,6	24,4	22,4	Positive	24,9	26,4	28,0	30,7	Positive	23,5	23,3	33,2	Positive

14	**10654**	1,22E+04	23,9	25,4	21,9	Positive	25,6	27,1	29,0	NA	Positive	25,6	25,0	31,9	Positive
15	**16849**	1,13E+04	24,0	25,0	21,0	Positive	25,3	26,0	28,3	39,3	Positive	25,5	24,5	28,5	Positive
16	**16140**	1,07E+04	24,1	25,3	24,0	Positive	25,8	27,1	30,1	NA	Positive	27,6	26,3	15,3	Positive
17	**17904**	3,14E+03	26,0	27,4	25,7	Positive	27,6	28,4	30,4	29,3	Positive	27,7	26,4	30,0	Positive
18	**16052**	1,87E+03	26,8	28,0	24,0	Positive	28,4	29,2	31,5	31,1	Positive	30,4	27,5	30,5	Positive
19	**15908**	1,06E+03	27,7	29,3	22,4	Positive	29,7	30,5	33,7	33,8	Positive	32,8	29,2	29,5	Positive
20	**17888**	8,29E+02	28,1	29,1	23,7	Positive	NA	29,8	32,3	29,8	Positive	29,8	28,7	30,4	Positive
21	**16961**	7,03E+02	28,3	31,3	25,1	Positive	29,9	30,3	33,3	30,4	Positive	32,7	28,5	29,4	Positive
22	**17097**	4,67E+02	28,9	29,5	23,7	Positive	32,7	33,4	36,1	NA	Positive	NA	30,8	32,3	Positive
23	**16080**	4,26E+02	29,1	30,4	23,5	Positive	30,8	32,3	34,2	33,3	Positive	31,3	33,5	NA	Positive
24	**16855**	3,78E+02	29,3	30,8	30,7	Positive	30,6	31,9	33,3	28,9	Positive	32,2	30,6	30,1	Positive
25	**17881**	3,78E+02	29,3	30,7	24,8	Positive	30,1	30,5	32,5	29,2	Positive	30,0	28,6	29,0	Positive
26	**16189**	3,53E+02	29,4	30,6	26,5	Positive	32,2	32,2	35,1	31,8	Positive	35,1	29,2	31,9	Positive
27	**15805**	3,50E+02	29,4	31,0	22,5	Positive	30,9	31,7	33,1	29,3	Positive	32,8	NA	NA	P.Positive
28	**13110**	3,01E+02	29,6	31,3	2,2	Positive	31,4	33,1	34,1	32,3	Positive	31,4	29,9	30,1	Positive
29	**15504**	2,59E+02	29,8	32,2	22,6	Positive	32,3	32,5	36,1	37,6	Positive	NA	31,8	32,1	Positive
30	**17897**	2,29E+02	30,0	31,8	24,8	Positive	31,6	32,3	34,5	29,5	Positive	34,2	30,0	29,4	Positive
31	**15985**	2,26E+02	30,1	31,4	26,3	Positive	31,7	33,2	35,9	32,3	Positive	33,6	30,4	30,4	Positive
32	**16003**	2,21E+02	30,1	33,0	20,4	Positive	33,9	33,7	36,1	35,7	Positive	NA	NA	NA	Invalid
33	**17892**	2,03E+02	30,2	33,1	24,3	Positive	32,4	33,2	35,8	33,2	Positive	NA	NA	29,4	Negative
34	**17089**	2,00E+02	30,2	31,4	25,5	Positive	39,2	37,9	41,9	NA	Positive	NA	33,3	NA	Positive
35	**17896**	1,83E+02	30,4	32,3	25,2	Positive	31,9	32,6	34,1	30,0	Positive	NA	NA	NA	Invalid
36	**16845**	1,79E+02	30,4	32,4	26,9	Positive	32,4	32,7	35,5	32,9	Positive	31,1	NA	23,1	P.Positive
37	**9243**	1,45E+02	30,7	33,2	19,1	Positive	33,3	34,6	37,2	36,1	Positive	NA	NA	NA	Invalid
38	**16104**	1,42E+02	30,8	34,5	27,0	Positive	NA	44,1	NA	NA	Positive	NA	NA	NA	Invalid
39	**15633**	9,04E+01	31,5	36,1	21,5	Positive	33,5	34,0	36,4	34,3	Positive	NA	NA	33,1	Negative
40	**16002**	8,98E+01	31,5	33,7	23,5	Positive	33,7	34,3	36,3	29,9	Positive	NA	NA	30,7	Negative
41	**17886**	7,67E+01	31,7	34,7	22,2	Positive	NA	34,5	NA	30,5	Positive	NA	NA	30,0	Negative
42	**17883**	7,56E+01	31,7	33,4	29,7	Positive	33,9	34,6	36,4	30,6	Positive	NA	32,0	29,0	Positive

43	**13034**	7,44E+01	31,8	36,7	21,7	Positive	33,2	34,2	37,2	32,4	Positive	NA	NA	NA	Invalid
44	**17905**	5,95E+01	32,1	33,7	27,5	Positive	33,6	33,8	36,7	30,3	Positive	NA	33,9	NA	Positive
45	**16054**	5,58E+01	32,2	33,8	24,0	Positive	33,6	34,2	37,1	31,6	Positive	NA	NA	31,0	Negative
46	**17889**	4,58E+01	32,5	35,4	25,1	Positive	34,2	33,8	37,2	31,1	Positive	NA	NA	31,3	Negative
47	**17887**	3,67E+01	32,9	35,3	27,3	Positive	34,4	35,4	39,1	29,9	Positive	NA	NA	29,9	Negative
48	**15983**	3,34E+01	33,0	34,3	26,2	Positive	33,9	35,1	37,6	33,2	Positive	NA	NA	29,5	Negative
49	**16117**	2,41E+01	33,5	36,4	26,7	Positive	34,0	35,4	37,0	31,7	Positive	NA	30,0	29,7	Positive
50	**17919**	1,83E+01	33,9	36,9	26,2	Positive	35,3	36,4	38,4	29,7	Positive	NA	NA	30,1	Negative
51	**10651**	1,28E+01	34,5	39,2	26,4	Positive	NA	NA	NA	29,9	Negative	NA	NA	NA	Invalid
52	**17882**	1,11E+01	34,7	37,9	27,5	Positive	36,1	37,6	39,0	31,7	Positive	NA	NA	30,6	Negative
53	**17893**	1,04E+01	34,8	36,1	28,1	Positive	35,5	36,5	38,3	30,1	Positive	34,9	NA	30,2	P.Positive
54	**13057**	8,63E+00	35,1	39,6	23,5	Positive	NA	37,8	38,0	31,0	Positive	NA	NA	NA	Invalid
55	**16009**	6,50E+00	35,5	39,4	26,4	Positive	NA	39,8	NA	28,6	Positive	NA	NA	28,3	Negative
56	**16125**	5,65E+00	35,7	38,7	27,4	Positive	36,8	36,0	NA	33,9	Positive	NA	NA	33,5	Negative
57	**16110**	5,15E+00	35,9	38,7	26,8	Positive	NA	NA	NA	31,3	Negative	NA	NA	NA	Invalid
58	**15505**	NA	NA	NA	24,7	Negative	36,7	36,0	38,3	32,1	Positive	NA	NA	30,6	Negative
59	**15515**	NA	NA	NA	22,9	Negative	37,2	37,3	NA	31,2	Positive	NA	NA	31,8	Negative
60	**16332**	NA	NA	NA	24,6	Negative	NA	NA	NA	31,5	Negative	NA	NA	31,7	Negative
61	**16333**	NA	NA	NA	25,7	Negative	NA	NA	NA	30,6	Negative	NA	NA	31,3	Negative
62	**16334**	NA	NA	NA	26,0	Negative	NA	NA	NA	32,2	Negative	NA	NA	31,7	Negative
63	**16335**	NA	NA	NA	24,7	Negative	NA	NA	NA	30,8	Negative	NA	NA	NA	Negative
64	**15512**	NA	NA	NA	22,0	Negative	NA	NA	NA	NA	Negative	NA	NA	NA	Negative
65	**15518**	NA	NA	NA	21,3	Negative	NA	NA	NA	NA	Negative	NA	NA	NA	Negative
66	**15519**	NA	NA	NA	21,4	Negative	NA	NA	NA	NA	Negative	NA	NA	NA	Negative
67	**15520**	NA	NA	NA	21,4	Negative	NA	NA	NA	NA	Negative	NA	NA	NA	Negative
68	**13174**	NA	NA	NA	23,9	Negative	NA	NA	NA	30,1	Negative	NA	NA	29,6	Negative
69	**13175**	NA	NA	NA	22,9	Negative	NA	NA	NA	NA	Negative	NA	NA	NA	Negative
70	**13183**	NA	NA	NA	25,7	Negative	NA	NA	NA	31,5	Negative	NA	NA	29,5	Negative
71	**13184**	NA	NA	NA	23,4	Negative	NA	NA	NA	32,6	Negative	NA	NA	27,9	Negative

72	**13185**	NA	NA	NA	25,5	Negative	NA	NA	NA	32,2	Negative	NA	NA	29,1	Negative
73	**13186**	NA	NA	NA	24,7	Negative	NA	NA	NA	33,9	Negative	NA	NA	29,1	Negative
74	**13187**	NA	NA	NA	21,1	Negative	NA	NA	NA	31,8	Negative	NA	NA	31,9	Negative
75	**15503**	NA	NA	NA	25,1	Negative	NA	NA	NA	30,1	Negative	NA	NA	28,6	Negative
76	**15506**	NA	NA	NA	26,0	Negative	NA	NA	NA	NA	Negative	NA	NA	NA	Negative
77	**15507**	NA	NA	NA	23,5	Negative	NA	NA	NA	30,4	Negative	NA	NA	28,9	Negative
78	**15508**	NA	NA	NA	24,6	Negative	NA	NA	NA	NA	Negative	NA	NA	NA	Negative
79	**15521**	NA	NA	NA	20,6	Negative	NA	NA	NA	NA	Negative	NA	NA	NA	Negative
80	**15524**	NA	NA	NA	20,6	Negative	NA	NA	NA	NA	Negative	NA	NA	23,4	Negative
81	**15525**	NA	NA	NA	19,2	Negative	NA	NA	NA	NA	Negative	NA	NA	34,4	Negative
82	**15510**	NA	NA	NA	21,4	Negative	NA	NA	NA	31,2	Negative	NA	NA	37,8	Negative
83	**15511**	NA	NA	NA	22,9	Negative	NA	NA	NA	30,0	Negative	NA	NA	29,8	Negative
84	**15514**	NA	NA	NA	22,0	Negative	NA	NA	NA	30,1	Negative	NA	NA	30,0	Negative
85	**15516**	NA	NA	NA	20,1	Negative	NA	NA	NA	31,8	Negative	NA	NA	30,3	Negative
86	**15517**	NA	NA	NA	19,8	Negative	NA	NA	NA	38,6	Negative	NA	NA	NA	Negative
87	**15522**	NA	NA	NA	21,2	Negative	NA	NA	NA	32,8	Negative	NA	NA	NA	Negative
88	**15523**	NA	NA	NA	21,1	Negative	NA	NA	NA	31,0	Negative	NA	NA	NA	Negative
89	**15526**	NA	NA	NA	21,1	Negative	NA	NA	NA	33,0	Negative	NA	NA	NA	Negative
